# Advanced paternal age effects in neurodevelopmental disorders—review of potential underlying mechanisms

**DOI:** 10.1038/tp.2016.294

**Published:** 2017-01-31

**Authors:** M Janecka, J Mill, M A Basson, A Goriely, H Spiers, A Reichenberg, L Schalkwyk, C Fernandes

**Affiliations:** 1 Social, Genetic and Developmental Psychiatry Centre, Institute of Psychiatry, Psychology and Neuroscience, King's College London, London, UK; 2 Department of Psychiatry, Icahn School of Medicine at Mount Sinai, New York, NY, USA; 3 University of Exeter Medical School, University of Exeter, Exeter, UK; 4 Department of Craniofacial and Stem Cell Biology, MRC Centre for Neurodevelopmental Disorders, King's College London, London, UK; 5 Weatherall Institute of Molecular Medicine, University of Oxford, Oxford, UK; 6 School of Biological Sciences, University of Essex, Colchester, UK

## Abstract

Multiple epidemiological studies suggest a relationship between advanced paternal age (APA) at conception and adverse neurodevelopmental outcomes in offspring, particularly with regard to increased risk for autism and schizophrenia. Conclusive evidence about how age-related changes in paternal gametes, or age-independent behavioral traits affect neural development is still lacking. Recent evidence suggests that the origins of APA effects are likely to be multidimensional, involving both inherited predisposition and *de novo* events. Here we provide a review of the epidemiological and molecular findings to date. Focusing on the latter, we present the evidence for genetic and epigenetic mechanisms underpinning the association between late fatherhood and disorder in offspring. We also discuss the limitations of the APA literature. We propose that different hypotheses relating to the origins of the APA effects are not mutually exclusive. Instead, multiple mechanisms likely contribute, reflecting the etiological complexity of neurodevelopmental disorders.

## Introduction

Epidemiological studies have provided extensive evidence for an association between advanced paternal age (APA) at conception and a series of negative outcomes in offspring. These adverse effects include Mendelian (single-gene) disorders (for example, Apert syndrome, achondroplasia^1, 2, 3^), as well as those with a more complex etiology (for example, autism, schizophrenia^4, 5^), together with birth complications^6, 7^ and nonclinical phenotypes (for example, IQ and academic achievement^8, 9^). The current review focuses on the relationship between APA and neurodevelopmental disorders, critically discussing evidence from both human and rodent studies. Although young fatherhood has also been linked with adverse outcomes in offspring, given that at least some aspects of these effects are likely underlain by factors that are different from those linked with APA,^10^ they are not discussed here (but see for example, refs 11,12).

We begin this review by outlining key epidemiological findings, and discussing the degree to which they might have been affected by ascertainment bias and quality of phenotyping. Next, we summarize the debate regarding *de novo* and inherited origins of the APA effects, which reflects the lack of consensus about whether effects on offspring behavior are caused by age-related changes in paternal spermatogonial stem cells, or age-independent behavioral traits associated with a delay or extension of fatherhood. We propose that the lack of unequivocal evidence in this matter suggests that both mechanisms likely contribute, to a varying degree in familial and sporadic cases. We suggest that better stratification of the samples, as well as parsing out maternal age effects, will be necessary to fully resolve this question.

The remaining part of this review focuses on the *de novo* hypotheses, including accumulation of genetic mutations—exacerbated by spermatogonial selection mechanisms—and epigenetic modifications. We present these hypotheses in detail, and discuss how well they have been supported by studies in humans and mouse models. Finally, we discuss the limitations of the APA epidemiological and molecular research more broadly.

Throughout our work, we emphasize that resolving the contribution of APA to complex, neurodevelopmental disorders will require the integration of ‘competing' hypotheses and extensive dialog between epidemiology and molecular biology. Molecular research should be guided by the patterns emerging from population-based research, and likewise its findings need to inform the design of the epidemiological studies.

## Epidemiological findings

### Schizophrenia

Schizophrenia was the first neuropsychiatric disorder to be linked with APA.^5^ Epidemiological data from multiple cohorts demonstrated that the risk of the disorder increases with paternal age at conception.^13, 14, 15, 16^ Although the magnitude of this effect differed between studies, there was a consensus about a relatively early start of these effects. The risk for the disorder was shown to be elevated already for offspring of fathers in their mid-to-late 30s, and to continue to increase together with paternal age. Men who were in their 40s at conception were found to be two to three times more likely to father a child with schizophrenia than those in their mid-to-late 20s, although these estimates vary considerably between the cohorts. A possible explanation for such discrepancies is heterogeneity between the composition of different cohorts, with samples enriched for sporadic cases likely producing inflated estimates of the APA effects. The disorder odds ratios associated with APA have been shown to be higher in female than in male offspring,^17^ however, a meta-analysis by Miller *et al.*^18^ did not confirm the presence of such effects.

Despite problems in obtaining precise estimates of the size of the paternal age effects, the link between APA and schizophrenia is robust across different cohorts and ethnic groups,^19, 20, 21^ and remains significant after controlling for possible confounds, including socio-economic status, paternal psychiatric morbidity and maternal age. Furthermore, Frans *et al.*^22^ showed that these effects are likely transgenerational—after controlling for parental ages, the age of maternal, but not paternal grandfather, was associated with a higher risk of schizophrenia in the Swedish population.

### Autism

The association between APA and increased risk of autism in offspring was initially reported by Reichenberg *et al.*^4^ and was subsequently replicated by other groups.^6, 9, 11, 12, 20, 23, 24, 25, 26, 27, 28, 29, 30^ As in schizophrenia, the association between APA and increased risk of autism is evident in the offspring of fathers in their mid-to-late 30s, with more advanced paternal ages associated with higher odds ratios for the disorder. Similarly as for schizophrenia, higher susceptibility of female offspring to the APA effects suggested by some groups^4, 31^ was not replicated in a multi-cohort study.^11^

Studies looking at transgenerational persistence of the APA–autism association yielded conflicting results, reporting both that the age of maternal grandmother (British Avon Longitudinal Study of Parents and Children^32, 33^) and maternal grandfather (Swedish sample^34^), is associated with higher risk of autism. Possible reason for inconsistency in the pattern of results was the different diagnostic definitions adopted in these studies, with the British Avon Longitudinal Study of Parents and Children study relying on a more heterogeneous cohort than the Swedish one.

The association between APA and both autism and schizophrenia is interesting given the phenotypic and genetic overlap between these disorders. They are both neurodevelopmental in origin and characterized by social impairment,^35^ delay in development of several cognitive and perceptual abilities, prepubertal decline^36^ and behavioral rigidity.^37^ Evidence for genetic correlation between these disorders was highlighted by Sullivan *et al.*,^38^ who showed that family history of both schizophrenia and bipolar disorder is a risk factor for autism in two independent cohorts. Similarly, diagnosis of autism early in life increases the odds of developing schizophrenia later in life,^39, 40^ and a study of genome-wide association data^41^ demonstrated genetic correlation between these disorders more directly. Taken together, this evidence suggests that autism and schizophrenia may be underlain by partially overlapping etiological factors, and therefore robustness of association between APA and autism and schizophrenia may hold important cues for discovering the biological pathways that are particularly vulnerable to the APA effects and that contribute to the co-heritability of the disorders.

## *De novo* vs inherited APA effects

Although the epidemiological association between APA and autism/schizophrenia is now well established, the underlying mechanisms remain equivocal. APA effects have been attributed to both (i) inherited genetic factors in couples where an older male becomes a father or (ii) *de novo* genetic changes in paternal gametes that arise as a consequence of ageing. Studies attempting to explore these hypotheses have used different family designs to look at systematic differences between men who delay or extend fatherhood and those who do not, compared the APA effects among sporadic and familial cases, and used animal models. As outlined below, the evidence from these studies suggests involvement of both inherited and *de novo* factors, an observation consistent with the complexity of the disorders in question.

The notion that the effects of APA on offspring could be mediated by inherited genetic factors is underpinned by a possibility of a genetic correlation between characteristics of couples with high paternal age and psychiatric disorders enriched in children of older fathers. One possibility is that behavioral traits of men who delay fatherhood, for example, social withdrawal or aloofness, are also associated with a higher genetic risk for autism or schizophrenia. Consequently, children born to men with gene variants predisposing them to delay reproduction (for example, due to social withdrawal) would be at a higher risk of developing these disorders regardless of the age at which their father conceives. It is also plausible that these inherited genetic risk factors come from the maternal, rather than paternal side. This entails that women who decide to have children with older men are themselves enriched for certain characteristics that influence the child's genetic liability for psychiatric disorders. Although paternal psychiatric diagnoses are usually controlled for in epidemiological studies of APA, maternal diagnoses and subclinical paternal and maternal traits often remain unaccounted for.

To date there has been little direct evidence of systematic differences in psychiatric liability between men who are old vs young at conception. Nilsen *et al.*^42^ showed that in a Norwegian sample, men who fathered their first child at an advanced age were characterized by more health problems and risky health behaviors. Although deterioration in health is expected to correlate with advancing age, whether these effects also influence offspring mental health outcomes remains unknown. Other studies have found no association between behavioral traits (rigidity, aloofness, anxiousness and hypersensitivity) and age at paternity, in either men or their female partners.^43, 44^

Considering solely the maternal effects, a study in a Finnish non-affective psychosis cohort found that females with schizophrenia were more likely to have a child with an older man, but this association was not replicated in a general birth cohort.^45^ Meta-analysis of multiple genome-wide association studies, however, suggested that higher age at maternity is associated with elevated genetic risk for schizophrenia.^46^ In the context of a very high correlation between parental ages (0.74; ref. 24), this suggests that inherited effects associated with paternal age are still likely conflated with those that arise due to maternal genetic predisposition. Future studies attempting to parse out these effects are therefore warranted (as discussed below, the same applies to maternal and paternal *de novo* contributions).

**Figure 1:**
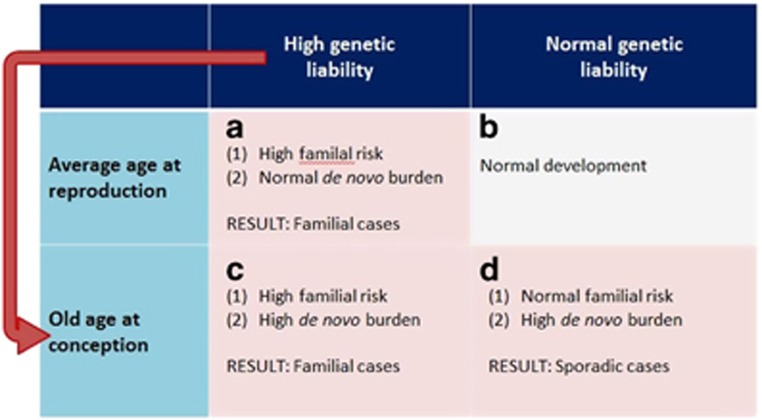
Different disorder etiologies among sporadic and familial cases. (**a**–**d**) Genetic liability or delayed reproduction by themselves increase the risk for, respectively, familial and sporadic forms of disorder (**a** and **d**, respectively). Given putative correlation between paternal genetic liability for psychiatric disorders and delayed reproduction, a substantial proportion of cases will arise due to a mixture of inherited and *de novo* factors (**c**).

Other groups have attempted to reconcile the *de novo* vs inherited origins of the APA effects by exploring whether these effects are more strongly associated with familial or sporadic forms of the disorder. The assumption underlying such designs is that if the inherited predisposition hypothesis was true, APA should be a risk factor for familial, but not sporadic disorders; on the other hand, the opposite would be observed if the APA effects arise mainly due to age-related accumulation of *de novo* factors. Studies relying on such design have yielded mixed evidence, showing that APA was associated with both schizophrenia only in sporadic cases^47^ and lack of such a pattern.^48^ Furthermore, Malaspina *et al.*^49^ reported that paternal age was higher in sporadic than familial cases, but Pulver *et al.*^50^ observed no such differences. Although studies of this kind have a great potential in shedding a light on the origins of the APA effects, they are still limited by the quality of phenotyping in relatives of the cases, and consequent confidence in classifying them as sporadic or familial. It also needs to be emphasized that conclusive evidence for higher paternal age in sporadic cases would not imply that APA effects are mediated solely by *de novo* mutations and are hence irrelevant for familial forms of the disorder. Likewise, the recognition of a significant proportion of sporadic cases in itself does not undermine the potential role of a polygenic component in the disorder etiology;^51^ therefore, dominant role of *de novo* effects is not an automatic corollary of lack of family history of the disorder. As illustrated in Figure 1, genetic liability likely interacts with age at conception, producing different patterns of familial/sporadic forms of the disorder in the population. Nevertheless, the degree to which these groups may be heterogeneous in terms of the genetic architecture underpinning their disorder remains to be investigated and clearly a dominant role for *de novo* effects is not synonymous of a lack of family history for the disorder.

A number of other family designs have been used to further the debate on inherited vs *de novo* effects of APA. Considering the effects of delaying fatherhood (age at first child) and advancing paternal age *per se,* several groups have found that only the former is associated with an increase in the risk of schizophrenia in offspring.^52, 53, 54^ In contrast, Hultman *et al.*^55^ showed that within families with siblings discordant for autism, the affected child is usually born later in the father's life, suggesting that age-dependent factors, rather than men's stable traits, explain the epidemiological association. As acknowledged before, such discrepancies in findings may result from cohort heterogeneity (Figure 1), or reflect a true difference in how APA mediates autism and schizophrenia in offspring.

Finally, some APA studies attempted to explore the contribution of *de novo* and inherited factors by using animal models. This was motivated by a possibility of strict experimental control over the timing of mating—thus ruling out paternal traits influencing the age at reproduction—afforded by such approach. Furthermore, control over the environmental variables (husbandry procedures, maternal age) and availability of inbred strains—which permit ruling out background genetic effects—can leave paternal age at conception as the only independent variable in the experiment. To our knowledge, all (published) APA animal studies have reported that advanced paternal age at conception is associated with changes in the behavioral domains relevant to autism and schizophrenia (social interaction and exploration,^56, 57, 58, 59^ motor coordination^60^ and pre-pulse inhibition^61^). Transgenerational persistence of these effects via the paternal line has also been shown by Sampino *et al.*,^59^ however, the design of their study did not permit assessing the effects acting through the maternal line (standardized age of the female breeders). Given the degree of experimental control over the factors other than the age-related genetic mutations in the paternal germline, these animal studies suggest that *de novo* effects likely have a role in mediating the APA effects. Nevertheless, there should be some caution in the interpretation of these patterns, given the limitations inherent in the use of these model organisms (see ‘Limitations of studies to date' section below). In short, eliminating the effects of familial factors—which likely have a role in humans^62^—could distort the relative importance of *de novo* factors. Second, the difference between rodent and human lifespan affects the rate at which these organisms age, limiting the translational potential of the findings.

In summary, disentangling the contribution of *de novo* and inherited factors to the APA effects is still challenging, and the evidence for their relative importance remains equivocal. We postulate that these effects likely interact. To fully resolve this issue, future studies will have to overcome the difficulties in reliable distinction between familial and sporadic cases, as well as establish, and account for, the contribution of maternal effects. Furthermore, to quantify the contribution of the *de novo* mutations to disorder etiology, their frequency and consequences in nonclinical samples will have to be better explored.^63, 64^ This will require an extensive cross-talk between epidemiological and molecular research. In the following sections, we focus on the literature exploring the role of the *de novo* effects, citing relevant evidence for age-related changes in paternal spermatogonial DNA, and corresponding effects in the offspring, in both humans and animal models.^56, 57, 59, 60, 61, 65, 66, 67, 68, 69, 70^ Further discussion regarding the inherited effects can be found in several recent publications.^52, 53, 54^

## Molecular findings

### *De novo* genetic mutations

The average number of *de novo* point mutations in a human newborn has been estimated at approximately 60–80.^71, 72^ Several studies suggested enrichment of these mutations in individuals with autism and schizophrenia,^66, 68, 73, 74^ although with a caveat of relying on samples with high proportion of cases from simplex families, naturally characterized by higher *de novo* burden. Owing to the primarily paternal origin of these *de novo* mutations and their accumulation with advancing paternal age,^66, 67, 69, 72, 73, 75^ they are suggested as the main mechanism underpinning the APA effects. Although the contribution of sporadic mutations to autism and schizophrenia is likely modest overall,^76, 77, 78, 79^ evidence suggests their relevance in a subset of cases.^66, 67, 68^

The baseline mutation rate in humans is around 1.1 × 10^-8^ per nucleotide per generation^80, 81, 82, 83^ (estimates from exome rather than whole-genome studies tend to be higher^84^). This entails the total number of *de novo* changes doubling every 16.5 years,^71, 85, 86^ highlighting the possible consequences of delaying fatherhood from the age of around 25 to 40. Although the rate of accumulation of these mutations has been questioned in the literature^87^ (and is likely variable between men^72, 84^), the overall age-related increase of mutagenic load in the gametes is now widely accepted, and attributed to the constantly dividing nature of the male germline. Owing to a risk of a DNA copy error associated with each of these replicative events, point mutations accumulate over time.^84, 85^

This stands in contrast to the dynamics of the female germline, where each oocyte is limited to 23 divisions, all of which occur before birth.^88^ The contribution of age-related accumulation of *de novo* mutations in female germline is only beginning to be acknowledged,^68, 75, 89^ with a recent study of 816 trios by Goldmann *et al.*^75^ suggesting increase of about 0.24 maternally transmitted *de novo* mutations per year (compared with 0.91 paternally transmitted; see for example, Kong *et al.,*^71^ who estimated the paternal increase to be 1.5–2 per year, without accounting for maternal effects and using ‘only' 78 family trios).^75, 90^ The molecular events underlying the maternal and paternal mutations associated with ageing are likely distinct,^75^ with a greater proportion of maternal mutations arising due to a failure of DNA double-strand break repair, rather than replication errors. The consensus still remains that the age-related increase in *de novo* mutations is larger in male than in female germline by over threefold,^75^ and that *de novo* point mutations in offspring are mainly of paternal origin.^66, 67, 69, 72, 73^ Nevertheless, given the parental age correlation discussed above, contribution of age-related mutational events in female germline might have not been sufficiently appreciated in the molecular APA research to date.

Regardless of parent-of-origin effects, the age-related accumulation of sporadic mutations could help account for the transgenerational persistence of the APA effects recorded in epidemiological research. In line with the rules of Mendelian inheritance, the mutagenic load in paternal spermatogonia would progressively accumulate in the following generations, increasing the risk of autism and schizophrenia in both children and grandchildren of men who were old at conception.

Nevertheless, it has been suggested that given the high polygenicity of both autism and schizophrenia,^76, 78, 79, 91^ additive effects of replication errors in the germline alone cannot explain the epidemiological patterns. Although these complex neurodevelopmental disorders remain a primary interest of the current review, important insights to address this issue were gained from the studies focusing on the APA-linked Mendelian disorders (for example, achondroplasia, Apert and Costello syndromes) and their causal mutations in the *FGFR2*, *FGFR3, PTPN11, HRAS* and *RET* genes. The mutation frequency at these loci in the male germline is much higher than that expected from the calculations above (10^−^^4^–10^−6^ vs expected 10^−9^–10^−7^ per nucleotide),^92, 93^ suggesting either hypermutability of certain loci, or additional mechanisms acting to propagate the cells carrying the mutation (selection model).

Although both the hotspot and selection models would result in a relative enrichment of cells with highly localized mutations, allelic distribution in the mutant cells,^94^ as well as replicated observation that these mutant cells tend to cluster in certain regions of the testes^92, 95, 96^ support the latter model (see also the review by Arnheim and Calabrese^93^). Statistical predictions, as well as lower probability of such clusters in young sperm donors^95^ further indicate it is unlikely that these clusters arose due to a mutation early in testis development, rather than selective expansion of cells with advantageous mutations. In the section below, we explain the principles of the selection model, and discuss its relevance to etiology of both Mendelian and complex disorders.

### Selection (‘selfish spermatogonial selection')

The ‘selfish spermatogonial selection' hypothesis postulates that stem cells with mutations at certain loci can gain selective advantage over non-mutated spermatogonial stem cells and expand clonally, leading to their relative enrichment in the germline.^82, 86, 97^ Although the mutational events giving rise to these effects are themselves rare, activating mutations in the genes involved in receptor tyrosine kinase (RTK)/RAS/mitogen-activated protein kinases (MAPK) pathway enhance the growth processes, leading to abnormal proliferation of spermatogonial stem cells that carry these mutations, via a process akin to oncogenesis (Maher *et al.*^96^ shows these occurrences in human testis).

Nevertheless, the deleterious disease phenotypes associated with these mutations render these mutations unlikely to be propagated through multiple generations, posing challenges in extrapolating the ‘selfish spermatogonial selection' hypothesis to complex, polygenic disorders like autism and schizophrenia.^79, 91, 98, 99^ To account for this, Goriely *et al.*^100^ suggested that other mutations—conferring less pronounced advantage in the testes—would be associated with milder variants and/or less penetrant disease phenotypes, thus reducing selection against them in subsequent generations and providing a source of heritable material. Similarly, other ‘passenger' (‘hitchhiker') mutations, could be co-inherited along with the selfish mutations driving the increased proliferation of spermatogonial cells, leading to their concurrent enrichment in paternal germline. This hypothesis is attractive because the genes that have so far been shown to be driving the selfish behavior of spermatogonial stem cells all belong to the RTK/RAS/MAPK pathway, which is one of the molecular modules known to be enriched in deleterious variants in neurodevelopmental disorders.^101, 102^ However, it remains to see whether experimental evidence support the idea that other mutations, aside from those that have been already characterized in the RTK/RAS/MAPK pathways display selfish properties. The advent of new techniques^96^ for visualizing the clonal expansions associated with such ‘selfish' *de novo* mutations promises to allow a better characterization of this process.

We suggest that further examination and refinement of the selfish selection hypothesis is likely to have a prominent role in elucidating the molecular *de novo* origins of APA effects. The proposed mechanism is consistent with the original hypothesis of increased burden of *de novo* mutations in male gametes, while also accounting for the problems with this basic proposal, like insufficiency of accumulation of these mutations to explain polygenic disorders like autism and schizophrenia. Furthermore, the disrupted regulation of specific growth factor pathways fits with the reported association between APA and some cancers in offspring,^97, 103, 104^ with close resemblance between clonal expansion of mutated germ cells and cell division in oncogenic tissues. Finally, the spermatogonial selection hypothesis would also account for the transgenerational persistence of the APA effects, reported in the epidemiological studies.^22, 59, 105^ Although more experimental evidence is still needed, there exists a plausible route through which the principles of the selection hypothesis could be applied to explain effects of APA on complex disorders like autism and schizophrenia.

### *De novo* epigenetic changes

Other mechanisms proposed to explain the association between APA and neuropsychiatric disorders in offspring include epigenetic effects—modifiable alterations to the DNA that occur independent of its sequence. These mechanisms may be of phenotypic importance given their potential influence on gene expression.^106^ Specific epigenetic alterations have been shown to be associated with psychiatric disorders, including autism and schizophrenia.^107, 108, 109, 110^ Most recent evidence reveals the role of the epigenetic marks in regulating offspring gene expression,^111^ suggesting that the paternally acquired factors affecting the offspring go beyond those conferred by the genome sequence alone.

### DNA methylation

In the context of health and disease, DNA methylation is the most extensively studied epigenetic modification, having a key role in gene regulation.^106^ Although traditionally regarded as a mechanism of transcriptional repression, DNA methylation can be associated with both increases and decreases in gene expression,^112^ and has recently been implicated in other genomic functions, including alternative splicing and promoter usage.^113^ DNA methylation marks are mitotically stable,^114, 115^ therefore modifications acquired over time in the gametes can be reliably maintained in the daughter cells, and thus accumulate over paternal lifespan in a manner similar to the process described above for *de novo* genetic mutations.^61, 116, 117^ However, persistence of these marks through meiotic divisions is likely very limited due to the two rounds of robust epigenetic reprogramming at fertilization.^118^ The first wave of global erasure of DNA methylation marks occurs before birth in embryonic primordial germ cells, and is followed by establishment of the new methylation patterns, differentially in male and female embryos. The second wave of erasure of these marks happens at fertilization, however, imprinted regions—discussed in detail below—escape this round of reprogramming, that is, the epigenetic marks in the imprinted regions present in the parental germline persist in the developing embryo.

Ageing process is associated with changes in the epigenetic landscape, and the levels of DNA methylation in multiple tissues can be used to reliably predict chronological age in humans.^119^ The involvement of such age-related epigenetic modifications in mediating APA effects was proposed by Malaspina in 2001,^5^ and since then the topic has received the attention from multiple research groups working on both animal^61, 120, 121^ and human samples.^122, 123, 124^ The overarching hypothesis behind this line of research is that age-related DNA methylation modifications in the paternal germline are retained at the zygotic stage, where they disrupt the normal developmental processes, resulting in an increased risk of disorders like autism and schizophrenia. Another possibility is that the changes in the DNA methylome affect mutability of certain loci, therefore, they do not need to persist transgenerationally to have an effect on offspring. Although such correlation between DNA methylation status and mutation rate is well established,^72^ this idea has received little attention in the context of APA research, and so the discussion below focuses on the former possibility, that is, effects of DNA methylation that do not entail genetic mutations.

Although several studies have identified seemingly APA-related modifications of the DNA methylome,^61, 120, 124^ these findings are yet to be replicated. Studies still lack consensus about the focal points of these changes (offspring tissue and/or DNA region-specificity) and functional consequences on development and behavior. The only study that analyzed age-related changes in the germline methylome in humans reported hypermethylation in the sperm of older males.^116^ However, these findings need to be taken with caution, given conflicting results from the rodent studies. Experiments in mice showed both hypo-^61^ and hypermethylation^116, 117^ of spermatogonial DNA at the global level, with similar changes in DNA methylation noted in brain tissue of offspring (ref. 61 showed hypo-, ref. 120 hypermethylation). Interrogation of candidate regions in rodent offspring revealed similarly inconsistent patterns.^120, 124^ These apparent discrepancies could be explained by (i) differences in methodologies used to quantify methylation in these studies; (ii) high degree of inter- and/or intra-individual variability in germline DNA methylation patterns;^125^ (iii) differences in the offspring type of tissue used for these analyses and (iv) uncertainty whether the changes occurred at paternally or maternally inherited alleles. It is, however, unlikely that these discrepancies arose as a result of (v) species-specific differences (only ref. 116 used human samples, with refs 61,117,120 all analyzing rodent samples).

Given that ageing is associated with unique epigenetic signatures,^119^ it is also possible that the findings described above reflect events that are ultimately unrelated to disease processes. Moreover, the age-related disruption of normal DNA methylation patterns in the gametes still cannot account for the father-to-offspring transmission of the APA effects. Paternal methylation marks are erased through active mechanisms^126^ before the blastocyst stage, with only the ones in the imprinted regions (reviewed below) remaining in the embryo's somatic cells. Therefore, the time window when the paternally acquired non-imprinted DNA methylation marks could affect offspring development is very limited. Although the reported differences between DNA methylome of offspring born to young and old fathers certainly warrant further consideration, we must emphasize that mechanistic understanding of putative father-to-offspring persistence of such effects is still lacking.

### Genomic imprinting

The DNA methylation hypothesis could be re-evaluated when focusing solely on the imprinted regions that escape the second round of epigenetic programming.^127^ Imprinting is a complex phenomenon whereby differential DNA methylation in paternal gametes is associated with monoallelic gene expression in offspring^128^ (nota bene the direction of this effect remains equivocal^129^). The imprints are established in the parental germline, inherited through the mitotic divisions post fertilization, erased in the fetal germline, and re-established later in an offspring sex-specific manner. The imprinting control regions often regulate monoallelic expression of a cluster of genes. Genes under such monoallelic regulation are found on both sex and autosomal chromosomes, and are characterized by age- and tissue-specific expression patterns.^130^ They have been shown to have a critical role in early development, including regulating placental formation and functioning,^131, 132^ and early brain development.^133^

Given that the parental imprints found in the germline can persist in the somatic cells of the offspring, and the developmentally important functions of imprinted genes, their involvement in mediating the APA effects is plausible. This would entail that age-related deficits in the maintenance of imprinted alleles in the paternal germline could lead to bi-allelic expression of the maternally expressed genes, resulting in abnormal development of offspring. A number of neurodevelopmental disorders have been previously attributed to errors in imprinting, including Prader–Willi syndrome,^134^ Angelman syndrome^135^ and Beckwith–Wiedemann syndrome.^136^

Although the possibility that APA effects may be mediated through errors in imprinting mechanisms has been under consideration for over a decade,^5, 123^ the experimental evidence for this hypothesis remains limited. One study in rodents showed that offspring of older fathers displayed differences in brain-expressed imprinted loci, with concurrent behavioral changes;^120^ however, it was not possible to unequivocally establish whether the reported effects were of paternal or maternal origin. To the best of our knowledge, there is still no research in humans in support of the involvement of imprinting errors in the APA effects.

Furthermore, the classical imprinting theory alone still cannot explain the transgenerational inheritance of the APA effect (grandpaternal age effects) reported in the epidemiological studies in humans^22, 34^ and research in rodents.^59^ The imprints escape the first wave of epigenetic reprogramming, and are erased and re-established in the offspring germline, thus precluding any grandfather-to-offspring passage. This does not rule out the contribution of imprinting errors to the APA effects, however, necessitates that other mechanisms that can be propagated through generations are also involved—a notion consistent with the hypothesis of multifactorial nature of paternal age effects. Although it has been suggested that transgenerational epigenetic inheritance of the imprints could still occur in the presence of a defect in the first wave of reprogramming,^137^ and this claim has been substantiated by several studies in rodents^118, 138^ and zebrafish,^139^ evidence in support of this notion is still lacking in humans. Generalizing these findings to the outbred populations—and to humans—still needs to be done with caution.

### Limitations of the studies to date

Elucidating the age-related changes in the germline that are causally involved in the APA effect on the risk of autism and schizophrenia will likely prove challenging—an inevitable corollary of complexity of the disorders in question. At present, we are unable to distinguish functionally important effects of ageing on the male germline from those that are just correlative, as well as the effects of age *per se* vs cumulative effects of other environmental exposures.

Furthermore, the discovery of such causal mechanisms is complicated by within-individual genetic heterogeneity of spermatogonial cells—resulting from chromosome recombination and different mutagenic signatures across the germline. Although studies of testicular tissue^96^ and sequencing of family trios^68, 71, 75^ can provide new insights into age-related dynamics of *de novo* mutations, there still remains a challenge of analyzing mutations in the particular cell that was used during fertilization of the affected individual. As suggested by Ségurel *et al.*,^84^ *de novo* mutations arising in parental germline or in the developing (post-zygotic) offspring cells cannot be reliably distinguished without looking at the subsequent (third) generation.

**Figure 2:**
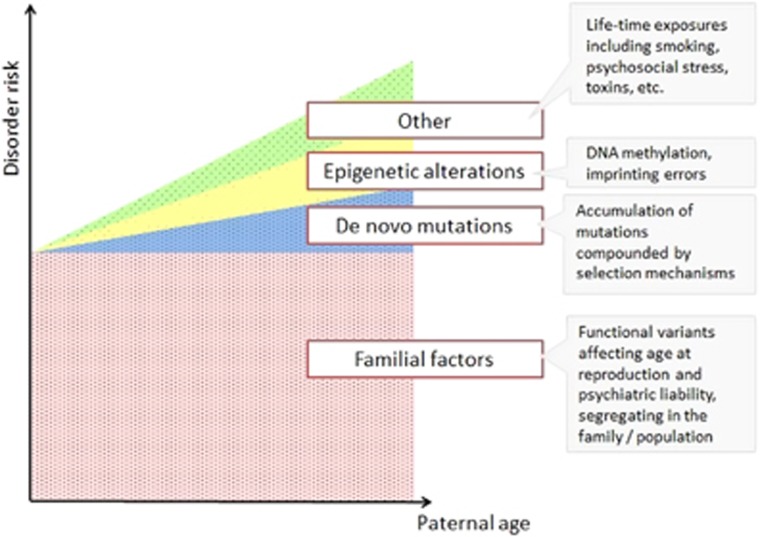
Multifactorial origins of disorder risk in offspring, in relation to paternal age at conception. The risk is underlain primarily by familial factors, except for in the case of extremely old fathers. The acquired factors likely involved DNMs (±selfish selection that is anticipated to load the genome with functional variants in pathways relevant to specific disease (RTK/RAS or other growth factors), epigenetic modifications or other factors. DNM, *de novo* mutation; RTK, receptor tyrosine kinase.

Moreover, studies to date tended to focus on a one chosen mechanism, exploring its contribution to the APA effects in isolation from other genomic and environmental events. Although such approaches are understandable from the feasibility perspective, the APA effects are likely multifactorial. As discussed above, autism and schizophrenia in the offspring of older fathers most likely arise due to a combination of inherited and *de novo* factors, with the latter further subdivided into genetic mutations, epigenetic alterations and other events related to lifetime exposures in parents (Figure 2). Although accumulation of *de novo* mutations has long been considered as the primary mediator of the APA effects, their role in autism and schizophrenia is relatively limited—recent exome sequencing study suggests that even among sporadic cases, only about 10% of diagnoses could be shown to arise partly due to *de novo* mutations.^66^

Additional evidence for these multifactorial origins of the APA effects comes from a recent population genetics study.^62^ The authors considered five possible genetic models underlying the APA effects, four of which focused on the *de novo*, and one on inherited factors. Among the *de novo* models, only the selfish selection model could explain more than 20% of increased risk of autism/schizophrenia in the offspring of older men, and only when assuming large magnitude of the clonal expansion of the mutated cells, with high proportion of the underlying mutations causally involved in the disorders in offspring. In contrast, the model assuming genetic correlation between age at paternity and psychiatric outcomes could fully explain the epidemiological patterns. Taking into account prior evidence for the role of the *de novo* events, the authors proposed that 50% of the APA effects are likely explained by inherited factors, 10–20% by *de novo* mutations, and the remainder by nongenetic factors. Although a degree of caution is warranted given that molecular validation of these results is still pending, the study should emphasize the multidimensional background of the APA effects.

Animal studies provide a strong support for the epidemiological and molecular findings from humans, however, their limitations in the context of APA research might not have been sufficiently considered. Beyond the widely acknowledged constraint in translation of behavioral phenotypes, differences in the mutation rates due to shorter lifespan in rodents^84^ and non-overlapping imprinted loci^140^ limit the construct validity of animal models of APA. Also, given that the effects in mice have only been investigated in models with a uniform genetic background (inbred strains), rodent studies have had no scope to testing the possibility that some men, because of their genetic make-up, will be more vulnerable to the effects of age on their germline. Evidence suggests more than twofold differences between men in their age-related increase in *de novo* mutations in their germline,^72^ with possible explanations for this variation including efficiency of the DNA repair system or germline methylation level.^84^ Even available outbred strains of laboratory mouse originate from random, and consequently partly inbred, colonies or are generated from a limited number of founders, which limits their genetic heterogeneity.^141^ Therefore, for the time being, animal models can provide only limited insights into the etiology of the APA effects in a genetically heterogeneous population. Finally, the role of environmental factors in the APA pathways is still unknown, and cannot be modelled in rodents. Given possible gene–environment correlation contributing to the APA effects, results from rodent studies have to be interpreted with caution.

Last but not least, the issue of what constitutes ‘advanced paternal age' has not received enough attention, which is particularly problematic in both human and animal studies treating paternal age as a categorical, rather than continuous variable. Owing to reduction in the sample size in the oldest paternal age categories, such designs render the findings more vulnerable to the effects of outliers, introducing considerable noise to the APA literature. A related issue is lack of control over the time of puberty—given higher rate of germ cells division (and therefore new mutations) after vs before adolescence, and variable age at the start of puberty,^142^ age at conception counted since puberty, rather than birth, is more relevant for APA research.^84^

## Conclusions

This review focused on the mechanisms underlying the association between APA and autism/schizophrenia in offspring. We postulate that inherited and *de novo* factors present complementary influences contributing to the epidemiological observations. Although these factors have been usually considered as competing hypotheses, the lack of unequivocal evidence to date suggests that likely both of them have a role.

Although the *de novo* mutations shown to accumulate with age in paternal sperm likely contribute to neurodevelopmental disorders in offspring, given the highly polygenic nature of these phenotypes, a substantial proportion of risk is still likely to arise owing to variants inherited from parents (Figure 2). Other factors that are also likely implicated include selection mechanisms, epigenetic alterations and accumulation of other lifetime exposures. Furthermore, maternal age effects will also have a role; studies suggesting correlation between risk of schizophrenia and age at conception^46^ and increase in *de novo* mutations in female germline^75^ suggest that maternal age effects likely contribute to both inherited and *de novo* risk factors.

We suggest that in order to fully resolve the APA effects, future research will need to use high sensitivity molecular techniques, such as single-cell sequencing of individual germ cells, combined with large human data sets with detailed epidemiological background information. Continuous dialog between epidemiological and molecular research will help refine the hypotheses investigated, and provide further insights into why older men are more likely to father a child with autism or schizophrenia, with implications for reproductive policies and genetic counseling.

Considering solely the risk for neuropsychiatric disorders, current evidence does not suggest that men should be discouraged from having a child at an older age. Given the low prevalence of these disorders at baseline, even a fivefold increase in the odds ratio under an assumption of strong *de novo* effects, would yield a low probability that the conceived child will have autism or schizophrenia as a result of higher paternal age. Nevertheless, more evidence is still needed to provide men and their partners with informed, reliable and accurate advice regarding the risks of delayed fatherhood.
